# Predictors of intracranial hemorrhage in neonatal patients on extracorporeal membrane oxygenation

**DOI:** 10.1038/s41598-023-46243-4

**Published:** 2023-11-07

**Authors:** Sara Wood, Riccardo Iacobelli, Sarah Kopfer, Caroline Lindblad, Eric Peter Thelin, Alexander Fletcher-Sandersjöö, Lars Mikael Broman

**Affiliations:** 1 ECMO Centre Karolinska, Intensive Care and Transport, Pediatric Perioperative Medicine and Intensive Care, Karolinska University Hospital, Akademiska Stråket 14, 171 76 Stockholm, Sweden; 2 Department of Clinical Neuroscience, Karolinska Institutet, Stockholm, Sweden; 3 Department of Neurosurgery, Uppsala University Hospital, Uppsala, Sweden; 4 Department of Medical Sciences, Uppsala University, Uppsala, Sweden; 5 Department of Neurology, Karolinska University Hospital, Stockholm, Sweden; 6 Department of Neurosurgery, Karolinska University Hospital, Stockholm, Sweden; 7 Department of Physiology and Pharmacology, Karolinska Institutet, Stockholm, Sweden

**Keywords:** Risk factors, Cerebrovascular disorders, Stroke, Neurology, Stroke, Cardiology, Cardiac device therapy, Brain injuries, Cerebrovascular disorders, Stroke

## Abstract

Extracorporeal membrane oxygenation (ECMO) is a life-supportive treatment in neonatal patients with refractory lung and/or heart failure. Intracranial hemorrhage (ICH) is a severe complication and reliable predictors are warranted. The aims of this study were to explore the incidence and possible predictors of ICH in ECMO-treated neonatal patients. We performed a single-center retrospective observational cohort study. Patients aged ≤ 28 days treated with ECMO between 2010 and 2018 were included. Exclusion criteria were ICH, ischemic stroke, cerebrovascular malformation before ECMO initiation or detected within 12 h of admission, ECMO treatment < 12 h, or prior treatment with ECMO at another facility > 12 h. The primary outcome was a CT-verified ICH. Logistic regression models were employed to identify possible predictors of the primary outcome. Of the 223 patients included, 29 (13%) developed an ICH during ECMO treatment. Thirty-day mortality was 59% in the ICH group and 16% in the non-ICH group (p < 0.0001). Lower gestational age (p < 0.01, odds ratio (OR) 0.96; 95%CI 0.94–0.98), and higher pre-ECMO lactate levels (p = 0.017, OR 1.1; 95%CI 1.01–1.18) were independently associated with increased risk of ICH-development. In the clinical setting, identification of risk factors and multimodal neuromonitoring could help initiate steps that lower the risk of ICH in these patients.

## Introduction

Extracorporeal membrane oxygenation (ECMO) is a method to ensure sufficient tissue oxygenation and perfusion in critically ill patients with refractory lung and/or heart failure when conventional intensive care is insufficient. However, the risk of complications such as infection, thrombosis, gaseous embolism, and hemorrhage is high^1,2^.

Intracranial hemorrhage (ICH) during ECMO is challenging to manage and is associated with high morbidity and mortality. Prior studies indicate an incidence between 9.9 and 28% in neonates, and a mortality rate of up to 57%^2–7^. Dalton et al. reported ICH in 22.5% of neonates treated on ECMO in eight centers with high inter-center variability regarding both thrombotic and bleeding events^2^. In a study of 32 neonates treated on ECMO for persistent pulmonary hypertension (PPHN), ICH found on head ultrasound was reported in 34% and was associated with lower fibrinogen levels and lower platelet count^3^. One-hundred-sixty neonatal and pediatric ECMO patients were screened with head ultrasound in a study by Carpenter et al., who found intracranial hemorrhage in 18% of the patients^4^. In 50 children with congenital heart disease treated on veno-arterial (VA) ECMO, Yang et al. reported findings of ICH on head ultrasound and/or computed tomography of the brain in 28% of patients. In neonatal patients, ECPR use and high procalcitonin levels were independently associated with ICH^5^. In a two-decade-old registry study by Hardart et al. (1999), ICH was present in 9.9% of 4550 neonatal ECMO patients. Gestational age, sepsis, acidosis, treatment with epinephrine, and coagulopathy were independent risk factors for ICH development^7^. In a similar work including 1398 prematurely born treated with ECMO, the same group reported ICH in 13% of cases. In these patients, gestational age, sepsis, acidosis, and treatment with sodium bicarbonate were independent predictors of ICH^6^.

Despite being important contributions to the field, these studies were limited by either low sample sizes, the combination of neonatal and pediatric patients into one entity, and/or included only a specific subset of ECMO patients, limiting their external validity. Moreover, ECMO treatment is continuously evolving, and reliable predictors of ICH in the neonate ECMO population need further assessment. This study aimed to further explore the incidence and possible predictors of ICH in ECMO-treated neonatal patients using single-center data from a high-volume ECMO center.

## Materials and methods

We performed a retrospective observational study at a high-volume ECMO ICU, offering ECMO support for all age groups. All patients 0–28 days old, regardless of gestational age, treated on ECMO between March 2010 and December 2018 were eligible for inclusion. The primary outcome was ICH, diagnosed by a computed tomography (CT) scan. Patients with ICH, ischemic stroke, or known cerebrovascular malformation upon admission or detected within 12 h of admission were excluded. To reduce the influence of precipitating events, i.e., prior brain lesions caused by other mechanisms than ECMO support or other ECMO support strategies, patients treated with ECMO < 12 h were excluded, as well as re-admitted patients and those treated with ECMO at another facility > 12 h before admission. The 12-h time point was chosen in accordance with previous studies from our center^8–10^.

### Patient management

Neonatal patients were cannulated using a surgical, open approach. For venovenous (VV) support, patients were cannulated via the right jugular vein (RJV) with a 13 French (Fr, outer diameter = Fr/3 mm) OriGen (OriGen Biomedical, Burladingen, Germany) dual-lumen cannula for support. For VA ECMO, an 8–12 Fr Bio-Medicus (Medtronic, Tolochenaz, Switzerland) lighthouse tip drainage cannula was placed via the RJV in the middle part of the right atrium. The return cannula was an 8–10 Fr Bio-Medicus (Medtronic) placed into the right carotid artery. A Medos HILITE 800LT membrane lung (Medos Medizintechnik, Stolberg, Germany; Xenios AG., Heilbronn, Germany) was used, as well as tubing with a surface coating for increased biocompatibility (Carmeda, Medtronic). Until 2011, CAPS roller pumps (Stöckert, Munich, Germany) were used. In 2012–2013 the roller pumps were successively phased out in favor of the Pedivas/CentriMag (Levitronix, Zurich, Switzerland) centrifugal pump console, which was used on 2 patients in 2013 and on all patients 2014 onwards. Anticoagulation was achieved by a continuous intravenous infusion of unfractionated heparin targeting an activated partial thromboplastin time (APTT) of 1.5–2 times the upper normal limit and monitored three times daily. Activated clotting time (target: 180–210 s) was assessed hourly for early detection of deviation of trend. During treatment, patients were in most cases awakened within the first days and a bedside ECMO specialist nurse regularly performed neurological checks, including brainstem reflexes and pupillary examinations. When neurological symptoms presented themselves (e.g., seizure, pupillary abnormalities, abnormal motor behavior, or decreased consciousness) a brain CT scan was always performed. Additional brain CT scans were also generally performed whenever a critically ill patient was referred for a thoracic or abdominal CT scan, even in the absence of neurological symptoms. Head ultrasound scans were sometimes used to screen for intracranial abnormalities. These were not performed to diagnose ICH or in patients with symptoms. If head ultrasound findings were pathological, CT scans were always performed to verify or negate such findings.

### Variables

The following data were collected: gestational age, gestational weight, sex, ICU diagnosis, pre-ECMO arterial blood gas analysis, pediatric index of mortality (PIM), ECMO configuration (VA or VV), extracranial thrombosis (venous and arterial thromboembolism), extracranial hemorrhage, and need for continuous renal replacement therapy (CRRT). Days on ECMO until ICH, days of ECMO support, and 30-day mortality were also collected. The primary outcome was a CT-verified ICH diagnosed during ECMO support. Clinical data were reviewed using the medical records software TakeCare (CompuGroup Medical Sweden AB, Farsta, Sweden), and imaging data were retrieved from the radiological management software Sectra Picture Archiving and Communication System (PACS) IDS7 (Sectra AB, Linköping, Sweden). Possible predicting variables were recorded until discharge or ICH detection. Patients with negative CT findings and patients with no CT imaging were regarded as not having developed an ICH, in accordance with previous studies^8,10,11^.

### Statistical analysis

Shapiro–Wilk's test was used to test for variable normality. Normally distributed continuous data are presented as mean (± SD), non-parametric continuous data as median (IQR), and categorical data as number and frequency (%). To determine possible predictors of the primary outcome, a univariable regression analysis was performed with ICH (dichotomized) as the dependent variable. Variables that showed a trend towards significance (p < 0.1) in the univariable regression were then added to a step-down multivariable logistic regression model to determine independent predictors of ICH. In the step-down model, the least significant factor was sequentially eliminated until only significant variables remained. For random missing data, the Markov chain Monte Carlo method (n = 5) was used for imputation before the multivariable analysis was performed on pooled data. The statistical significance level was set to p < 0.05. Analyses were conducted using SPSS (version 27.0, IBM Corp, Armonk, New York) and graphs were created using the ggplot2 package in R with the interface RStudio (RStudio, Boston, MA, USA).

### Ethics approval and consent to participate

The study was performed in accordance with the declaration of Helsinki and approved by the Regional Ethical Review Board in Stockholm, Sweden (DNR: 2018/830-31). The requirement for informed consent from the study subjects was waived by the Regional Ethical Review Board in Stockholm, Sweden (DNR: 2018/830-31) due to the retrospective study design.

## Results

### Baseline data

**Figure 1:**
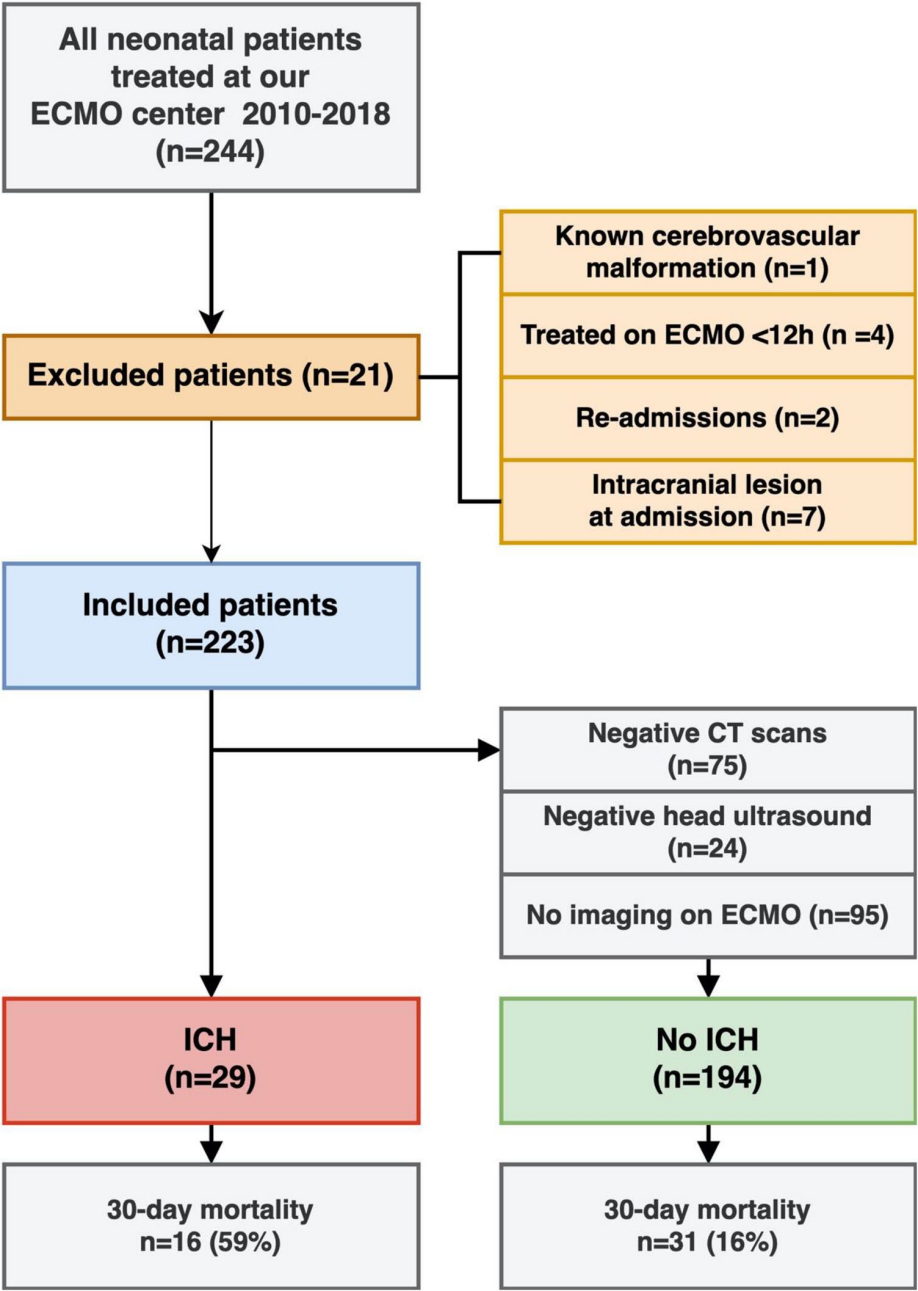
Flow chart showing patient selection, stratification, and mortality. All 29 ICH were diagnosed with a brain CT scan. *ECMO* extracorporeal membrane oxygenation, *ICH* intracranial hemorrhage.

Between 2010 and 2018, 244 neonatal patients were admitted for ECMO treatment. Of these, 21 were excluded and the remaining 223 were included in the study (Fig. 1).

Patient characteristics are presented in Table 1. Most patients (n = 160, 72%) were treated with VA ECMO, with the remaining 63 treated with VV support. The most common ECMO indication was meconium aspiration syndrome (MAS, n = 84, 38%), followed by congenital diaphragmatic hernia (CDH, n = 39, 17%). Median gestational age was 40 weeks + 0 days (IQR: 38 weeks + 2 days to 41 + 1) in the non-ICH group and 38 + 1 (35 + 0 to 39 + 4) (p < 0.001) in the ICH patients. Missing data was present in eight variables, amounting to 2.9% of all included values. PIM score (31.4% missing), lactate levels (16.6%), pO_2_ (9%), and pCO_2_ (7.2%) were variables with > 5% missing data.

**Table 1:** Demographics, birth characteristics, indication, and ECMO-mode of the non-ICH group and ICH group respectively.

	Non-ICH group (n = 194)	ICH group (n = 29)
Male sex	112 (58%)	14 (48%)
Gestational weight (kg)	3.5 (3.0–3.9)	3.2 (2.8–3.7)
Gestational age (days)	280 (268–288)	267 (245–277)
ECMO indications		
Heart failure	18 (9%)	2 (6.9%)
Respiratory failure	23 (12%)	6 (21%)
CDH	32 (16%)	7 (24%)
ECPR	2 (1%)	1 (3%)
MAS	82 (42%)	2 (7%)
PPHN	21 (11%)	4 (14%)
Sepsis^a^	26 (13%)	7 (24%)
VA ECMO mode	134 (69%)	26 (90%)
PIM (EMR %)	31 (19–55)	52 (34–67)
Pre-ECMO ABG		
pH	7.2 (7.1–7.3)	7.1 (7.0–7.2)
*p*CO_2_	7.6 (6.0–9.8)	8.6 (6.1–12.2)
*p*O_2_	4.7 (3.4–6.6)	4.6 (3.0–5.9)
Lactate	4.2 (1.7–7.3)	5.4 (2.9–10.8)
CRRT	134 (69%)	26 (90%)
Extracranial thrombosis	14 (7%)	3 (10%)
Extracranial hemorrhage	49 (25%)	11 (38%)
Days on ECMO	6 (4–10)	7 (4–19)
Days to ICH detection	–	2 (1–4)
30-day mortality	31 (16%)	17 (59%)

Continuous variables presented as median (IQR) and categorical variables presented as number (percentages).

*ICH* intracranial hemorrhage, *CDH* congenital diaphragmatic hernia, *ECPR* extracorporeal cardiopulmonary resuscitation, *MAS* meconium aspiration syndrome, *PPHN* persistent pulmonary hypertension of the newborn, *VA* venoarterial, *VV* venovenous, *NA* not applicable.

^a^Including septic shock.

### ICH and patient outcome

**Figure 2:**
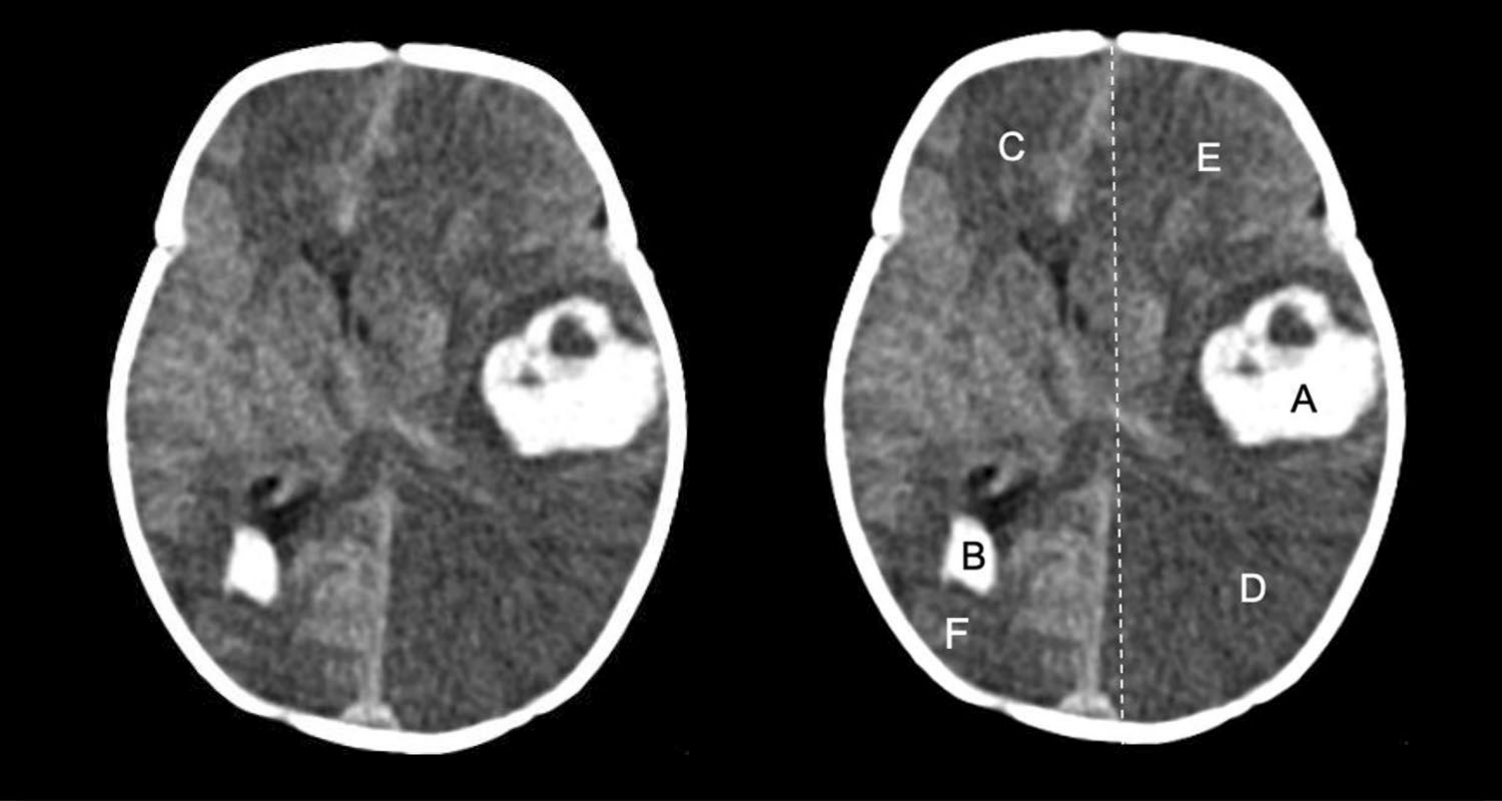
CT-scan showing a neonatal patient who developed a large intracerebral hematoma (**A**) with intraventricular extension (**B**), significant mass effect (mid-line depicted as a dotted line), and multiple ischemic lesion (**C–F**) during ECMO treatment. The original image is shown on the left.

One hundred four patients (47%) underwent at least one brain CT scan during their ECMO treatment. Out of all 223 included patients, 29 (13%) were diagnosed with an ICH. Of the 119 patients who did not undergo CT scanning, 24 patients had a negative head ultrasound at some time during their treatment. This leaves 95 (43%) patients without radiological data (Fig. 1). An example of a brain CT scan with ICH during ECMO is displayed in Fig. 2.

**Figure 3:**
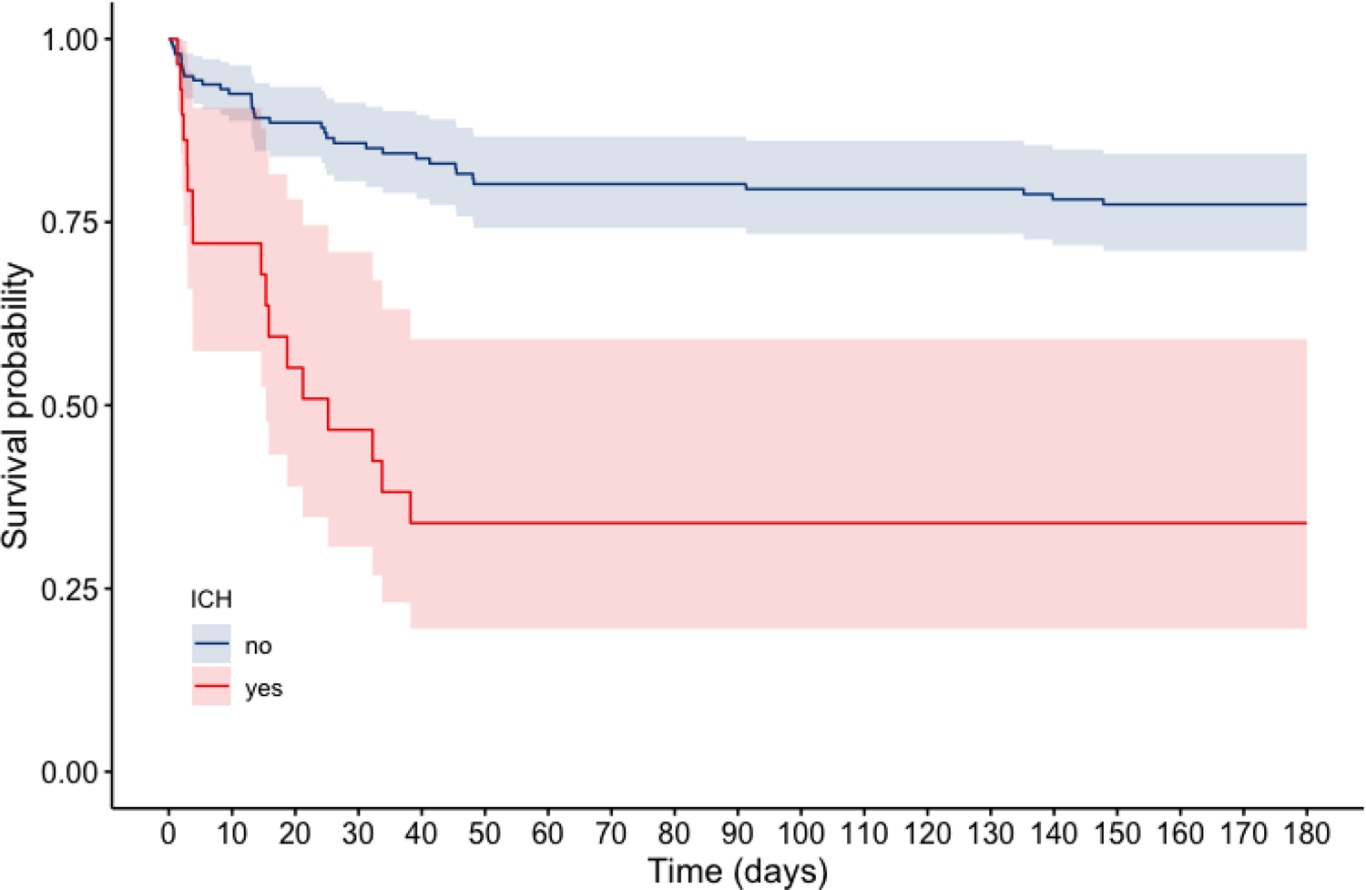
Kaplan–Meier plot showing 180-day survival stratified by ICH-status. Time is expressed in days after ECMO initiation. The shaded area shows a 95% confidence interval.

Most patients suffered intraparenchymal/intracerebral hemorrhages (52%), and twenty patients (69%) had a combination of two or more types of hemorrhages (Supplementary Table 1). The ICH patients had higher 30-day mortality compared to non-ICH patients (59% vs. 16%, p < 0.001). Survival probability is shown in a Kaplan–Meier model (Fig. 3).

### Predictors of ICH

In the univariable logistic regression predicting ICH development, VA ECMO mode (odds ratio (OR) 3.88 (95%CI 1.13–13.3), p = 0.03), lower gestational age (OR 0.96 (0.94–0.98), p < 0.01), and CRRT (OR 3.88 (1.13–13.3), p = 0.03), were significantly associated with ICH development. In the step-down multivariable model, lower gestational age (OR 1.04 (1.02–1.06), p < 0.01), and higher pre-ECMO ABG lactate levels (OR 1.1 (1.02–1.18), p = 0.017) were independent predictors of ICH development.

### Longitudinal laboratory analysis

**Figure 4:**
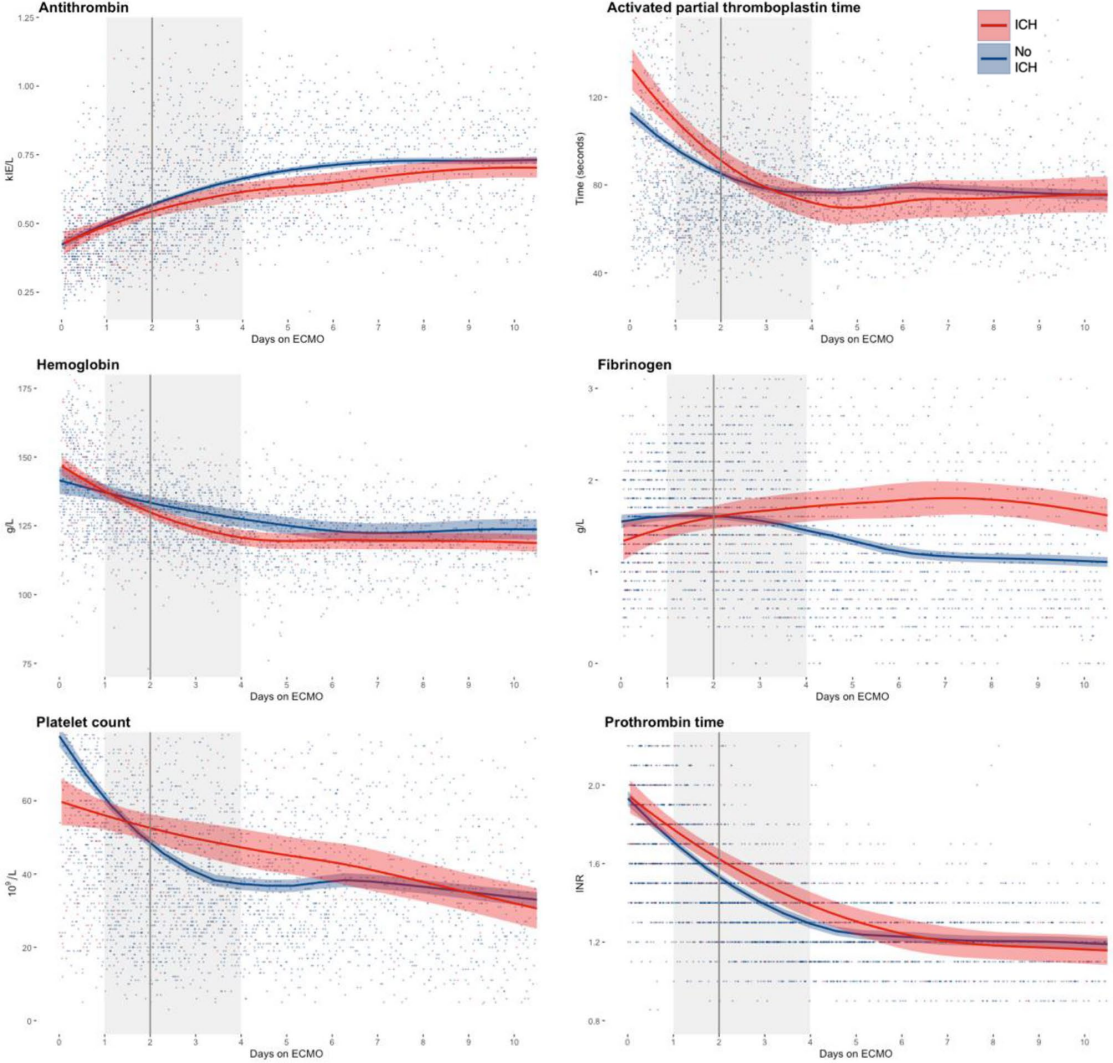
Scatterplots of hemostatic data depicted longitudinally and stratified according to ICH status. Patient included till detection of ICH or to time of explantation of ECMO. The lines indicate LOWESS curves and the shaded area surrounding them is the 95% confidence interval. The vertical black line represents the median time to ICH diagnosis (2 days), and the shaded area represents the interquartile range (1–4 days). *ECMO* extracorporeal membrane oxygenation, *ICH* intracranial hemorrhage, *INR* international normalized ratio. The graph was created using ggplot 2 (Wickham H. ggplot2: Elegant Graphics for Data Analysis, 2016. NY: Springer-Verlag).

Longitudinal coagulation biochemistry is summarized in Fig. 4. Patients who suffered an ICH displayed a lower platelet count and higher APTT after the commencement of ECMO.

## Discussion

This observational cohort study presents the largest single-center observational study of ICH predictors in neonatal patients on ECMO and corroborates findings that may be important for future patient management and study design. Out of 223 patients, 13% developed ICH. The 30-day mortality in the ICH group was 59%, as compared to 16% in the non-ICH group. Lower gestational age and higher pre-ECMO plasma lactate were independent predictors of ICH development.

Our study reports an incidence of 13%, which is a lower rate compared to some of the previously reported^2–5^, and slightly higher than in the retrospective study performed by Hardart et al.^7^. Of note, 53% of patients in our study did not undergo any CT during treatment. The incidence amongst patients with a brain CT was 28%. Moreover, 24 of these patients had a negative head ultrasound, leaving only 43% of our included patients with no neuroimaging. According to Elkhunovic et al., head ultrasound has a very high specificity for ICH (99%)^12^. Sirgiovanni et al. and Lidegran et al. have previously described that many cases of ICH on ECMO may pass unnoticed due to lack of neuroimaging^13,14^. The true incidence of ICH may thus be higher than our findings suggests, and may also explain why the incidence of ICH during ECMO treatment varies vastly between studies^2–4^. It is unclear if an incidentally found ICH is clinically relevant as the long-term effects of undetected ICH are not extensively studied. Bulas et al. described that the severity of cerebral insult during treatment correlated to a neuropsychological deficit, diminished intellectual status, and pre-academic shortfalls at a 5-year follow-up^15^. Even mild injuries may lead to an increased risk of neurophysiological deficit^15^, which further highlights the importance of neuroimaging. These lesions may impair the development of the individual child. An extensive multimodal neurological monitoring of these patients (continuous EEG, scheduled head ultrasound, serial S-100B samples) may help select neonates with subclinical symptoms during ECMO. However, conclusions about the benefits of additional neuromonitoring cannot be drawn from this study. Nevertheless, the 30-day mortality in patients with no neuroimaging was only 5% in our study. Therefore, we believe that asymptomatic hemorrhages likely have limited clinical significance in these critical care settings.

Lower gestational age was independently associated with a higher risk of ICH development. Similar findings have been previously reported by Hardart et al. in a registry-based study of 1,524 neonates with gestational age (GA) < 37 weeks. In the prematurely born (GA < 32 weeks), GA was independently associated with the development of ICH, which occurred in 26% of the population^6^. These findings may not be a surprise after the publication by Bartlett et al. (1977) who found ICH in 89% of the prematurely born before GA 35 weeks^16^. Gestational age < 34 weeks has since been regarded as a strong relative contraindication for treatment with ECMO. Lately, this has been challenged in a recent review by Mesas Burgos et al.^17^. As stated in their review, ECMO treatment has improved significantly over the past decades, with a lower rate of ICH complication and higher survival in early premature patients. Our study includes neonates from 2010 to 2018, furthermore technological advancement and increased knowledge may have led to safer management of the ECMO circuit and decreased rate of complications over the course of the study period. It is possible that the management of pre-term patients on ECMO has improved, and borders may shift in the future. Nonetheless, these patients may still be at higher risk for ICH development, as the immature vasculature and frail regulation of cerebral perfusion in the pre-term infant are susceptible to this complication^18^. Therefore, it may be wise to apply a generous radiological examination policy in these patients and, whenever possible, advocate for earlier weaning from ECMO and APTT range in the lower targeted interval.

Lactate levels prior to ECMO initiation were higher in ICH patients and were independently associated with ICH development during ECMO support, with an OR of 1.1, (1.02–1.18). This implies that each 1 mmol/l increase in lactate increases the odds of ICH by 10%. Lactate levels may serve as a surrogate for disease severity and may be associated with a more pronounced coagulopathy at ECMO initiation. The correlation between high lactate levels upon ECMO initiation and ICH has previously been described by Grayck et al., who studied 82 neonatal ECMO patients and found higher lactate levels in neonates that developed ICH and In non-survivors^19^. High lactate levels compromise the coagulation system and if uncorrected, this impairment may contribute to ICH progression in ECMO patients^20^. Hyperlactatemia in sepsis and disseminated intravascular coagulation are associated with impairment of activated fibrinolysis and coagulation disturbances that may contribute to a worse outcome^21^. Furthermore, in a study by Goswami et al., metabolic acidosis was associated with intraventricular hemorrhage in premature neonatal patients^22^. High lactate levels and acidosis may impair coagulation at the beginning of ECMO treatment, possibly facilitating ICH development.

Continuous renal replacement therapy was a predictor of ICH development in the univariable analysis, with an OR of almost 4. Patients on ECMO often require CRRT due to acute kidney injury (AKI). Renal regulation of hypertension and coagulation influence the development of intraventricular hemorrhage (IVH), and IVH itself may cause acidosis and hypotension, which in turn may cause AKI^23^. These patients are critically ill and likely to suffer other organ dysfunctions, which may explain a higher rate of complications. Therefore, it is difficult to draw conclusions on causality from our findings.

VA ECMO mode was also significantly associated with an increased risk of ICH in univariable analysis (OR 3.88). VA ECMO has previously been linked to unfavorable neurological complications in neonatal ECMO patients^10,24,25^. This may be explained by the effects of carotid ligation during cannulation, which was used in 94% of our VA ECMO patients, causing changes and slowing of cerebral blood flow. Moreover, these patients are generally sicker and require more intense support than VV patients, which may explain both the association with ICH and its influence on survival. Another contributing factor may be the increased hemodynamic instability often seen in patients requiring VA support already at commencement^25^. Furthermore, VA ECMO may be associated with brain infarction, as previous studies at our center suggest, and it is possible that a significant amount of ICHs are caused by a hemorrhagic transformation of brain infarctions, which has previously shown an association with VA ECMO and is a common complication in adults^8,10^.

In our cohort, nine (31%) patients had both an ICH and brain infarction on CT imaging. Brain infarction may precede ICH in these patients though it is impossible to draw any further conclusions regarding causality in this study, as only one patient had a brain infarction diagnosed before the ICH. The median time to ICH was also 2 days, making it hard to assess for any ischemic events prior to ICH. This suggests that neonates are more susceptible to ICH development at ECMO initiation or shortly thereafter when they may have been subjected to prolonged ischemia or hypercarbia. Hypercarbia and rapid restitution of pCO_2_ after commencement of ECMO is a recent but today well-known risk factor for neurological complications, including ICH^26^. However, the recovery and trajectory of paCO_2_ were not the scope of this study and can only be commented on from the observation that paCO_2_ was 1 kPa (7.5 mmHg) higher before ECMO start in the ICH group compared to the non-ICH patients. It is plausible that these patients may have been lowered more hastily as compared to non-ICH, as they had slightly higher levels before the commencement of treatment. On the other hand, reducing paCO_2_ in two hyper-carbic groups with a net difference of 1 kPa would unlikely differ much in fractional decrease where the target for all patients is upper normal range, i.e., 5.3–6 kPa. Nonetheless, data on pCO2 was not collected in this study after ECMO commencement, and accordingly, this could not be analyzed. The univariable analysis did not promote pre-ECMO paCO_2_ per se as a risk factor for ICH.

Graphically presented laboratory data over time show that ICH patients have a higher APTT, lower platelet count, a trend towards lower fibrinogen, lower hemoglobin, and higher prothrombin time in patients with ICH, prior to the median time to event. These changes may be explained by innate coagulopathy associated with ECMO support, the underlying pathology, and anticoagulants. The artificial membranes trigger an inflammation cascade and platelet and red blood cell activation, mainly through the activation of factor XI and the contact system, which eventually leads to a depletion of platelets and coagulation factors^27,28^. Hemoglobin is also depleted during ECMO due to continuous small blood losses and lower levels of hemoglobin are associated with a greater loss of platelets and coagulation factors^28^. It is plausible that patients with fatal hemorrhagic or thrombo-embolic complications exhibit a more pronounced coagulopathy, however, the driver(s) of this might be the severity of the disease itself and/or a complex combination of treatments, and management of anticoagulation. Coagulation monitoring in neonates on ECMO is still challenging, and APTT does not account for platelet function. In older studies, it was deemed to be a reliable monitor of heparin management in pediatric patients on ECMO with high values correlating to hemorrhagic complications^29^. However, newer studies suggest that the relationship between bleeding and APTT is not clear, and isolated testing of APTT is suboptimal, as it does not account for platelet function or thrombin generation^30^. Low plasma fibrinogen and platelet count have earlier been shown to predict ICH^3^. In contrast, a study by Anton-Martin et al. suggested that there was no association between laboratory coagulation parameters (activated clotting time, partial thromboplastin time, anti-factor Xa, platelet count, prothrombin time, fibrinogen, d-dimer) and ICH development^31^. In the current material, the median time to recognition of an ICH on ECMO was 2 days. Fibrinogen levels did not differ significantly between groups. Lower platelet count is consistent with previous findings by Doymaz et al., and studies on adults^3,9,32^.

### Limitations

The major limitation of this report relates to the diagnostics of ICH. CT scans were, for numerous reasons (as low as reasonably achievable—ALARA principle^33^), not performed routinely upon admission to the ECMO ICU. It is plausible that some patients may have developed subclinical ICH before ECMO treatment initiation. Moreover, patients who did not undergo CT scans were categorized as not having developed an ICH, which may have led to an underestimation of the true incidence of ICH in this cohort. However, this is consistent with previously published studies^8–10^. Furthermore, we believe that missed/subclinical ICHs have unclear clinical relevance in the acute phase (the 30-day mortality was only 5% in patients with no CT, as mentioned previously) and including all patients (no-CT patients as well), most accurately reflect the clinical settings and the neonatal ECMO population. Gestational age was analyzed as a continuous variable, rather than separating pre- and post-term neonates. The inherent differences in neurological complications may differ between these groups. At the beginning of the study, roller pumps were used and phased out in favor of centrifugal pumps. The study database does not have the granularity of pump manufacturer. As some patients treated at our center reside outside of Sweden, we had no access to data on patients after transport to their home country with ongoing ECMO support. In total, 52 patients (23%) were lost to follow-up due to transfer to other regions or countries after decannulation from ECMO. Because patients are transported from different regions or from abroad to our center, there was a high rate of missing data. Multiple testing was performed, which introduces a risk for type I errors. We did not adjust for multiple comparisons as this study was only hypothesis-generating. Even though most of the predictors identified are non-modifiable during ECMO support, they need to be externally validated before definitive clinical conclusions can be drawn. Nevertheless, awareness of risk factors increases the chance for recognition of at-risk patients and individualized management of anticoagulation and neuromonitoring.

## Conclusions

ICH is a frequent complication in neonatal patients treated with ECMO and associated with increased mortality. Lower gestational age and higher pre-ECMO lactate levels were independent predictors of ICH-development. In the clinical setting, identification of risk factors and multimodal neuromonitoring could help initiate steps that lower the risk of ICH in these patients.

### Supplementary Information

Supplementary Table 1.

## Data Availability

The datasets generated for quality assurance from patient charts during and/or analyzed during the current study are not made public to ensure professional secrecy but are available from the corresponding author on reasonable request.

## Abbreviations

ABG: Arterial blood gas
AKI: Acute kidney injury
APTT: Activated partial thromboplastin time
CDH: Congenital diaphragmatic hernia
CRRT: Continuous renal replacement therapy
ECMO: Extracorporeal membrane oxygenation
ECPR: Extracorporeal cardiopulmonary resuscitation
ICH: Intracranial hemorrhage
ICU: Intensive care unit
MAS: Meconium aspiration syndrome
PIM: Pediatric mortality index
PPHN: Persistent pulmonary hypertension of the newborn
RJV: Right jugular vein
VA: Venoarterial
VV: Venovenous
