# Supplementary material for: Predictors of intracranial hemorrhage in neonatal patients on extracorporeal membrane oxygenation
Source: Sci Rep. 2023 Nov 7;13:19249. doi: 10.1038/s41598-023-46243-4 (PMC10630488; doi:10.1038/s41598-023-46243-4)
Supplement: Supplementary file 1 — Supplementary Table 1. [file 41598_2023_46243_MOESM1_ESM.docx]

**Supplementary Table 1.** Intracranial hemorrhage characteristics

|  | **ICH group (n=29)** |
| --- | --- |
| Intracerebral hemorrhage | 15 (52%) |
| Intraventricular hemorrhage | 10 (34%) |
| Subarachnoid hemorrhage | 6 (21%) |
| Subdural hemorrhage | 8 (28%) |

Types of intracranial hemorrhage found in neonates during the studied period. Twenty (69%) patients had ≥ 2 types of hemorrhages simultaneously.
